# Ideotype Population Exploration: Growth, Photosynthesis, and Yield Components at Different Planting Densities in Winter Oilseed Rape (*Brassica napus* L.)

**DOI:** 10.1371/journal.pone.0114232

**Published:** 2014-12-17

**Authors:** Ni Ma, Jinzhan Yuan, Ming Li, Jun Li, Liyan Zhang, Lixin Liu, Muhammad Shahbaz Naeem, Chunlei Zhang

**Affiliations:** 1 Oil Crops Research Institute Chinese Academy of Agricultural Science, Key Laboratory of Oil Crop Biology of the Ministry of Agriculture, Key Laboratory of Crop Cultivation and Physiology, Ministry of Agriculture, Wuhan, China; 2 Key Laboratory of Crop Ecophysiology and Farming System in the Middle Reaches of the Yangtze River, Ministry of Agriculture, Huazhong Agricultural University, Wuhan, China; Huazhong university of Science and Technology, China

## Abstract

Rapeseed is one of the most important edible oil crops in the world and the seed yield has lagged behind the increasing demand driven by population growth. Winter oilseed rape (*Brassica napus* L.) is widely cultivated with relatively low yield in China, so it is necessary to find the strategies to improve the expression of yield potential. Planting density has great effects on seed yield of crops. Hence, field experiments were conducted in Wuhan in the Yangtze River basin with one conventional variety (Zhongshuang 11, ZS11) and one hybrid variety (Huayouza 9, HYZ9) at five planting densities (27.0×10^4^, 37.5×10^4^, 48.0×10^4^, 58.5×10^4^, 69.0×10^4^ plants ha^–1^) during 2010–2012 to investigate the yield components. The physiological traits for high-yield and normal-yield populations were measured during 2011–2013. Our results indicated that planting densities of 58.5×10^4^ plants ha^–1^ in ZS11 and 48.0×10^4^ plants ha^–1^ in HYZ9 have significantly higher yield compared with the density of 27.0×10^4^ plants ha^–1^for both varieties. The ideal silique numbers for ZS11 and HYZ9 were ∼0.9×10^4^ (n m^–2^) and ∼1×10^4^ (n m^-2^), respectively, and ideal primary branches for ZS11 and HYZ9 were ∼250 (n m^–2^) and ∼300 (n m^–2^), respectively. The highest leaf area index (LAI) and silique wall area index (SAI) was ∼5.0 and 7.0, respectively. Moreover, higher leaf net photosynthetic rate (Pn) and water use efficiency (WUE) were observed in the high-yield populations. A significantly higher level of silique wall photosynthesis and rapid dry matter accumulation were supposed to result in the maximum seed yield. Our results suggest that increasing the planting density within certain range is a feasible approach for higher seed yield in winter rapeseed in China.

## Introduction

Rapeseed is cultivated worldwide and plays an important role in guaranteeing an adequate food supply. Moreover, the increasing demand is fueled by its growing use as a renewable energy source [1]–[2]. In recent years, the yield is insufficient to meet the increasing demands, especially in China [3]. Winter oilseed rape (*Brassica napus* L.) is widely planted in the Yangtze River region, accounting for 89% of total rapeseed yields in China [3]–[4], whereas the yield per unit area decreased over past few years [5]. Therefore, it is necessary to find the strategies to improve the expression of yield potential.

The crop yield is influenced by the crop species, environmental conditions and agronomic factors [6]–[7]. Planting density is an important crop management that affects the seed yield [7]–[10]. High planting density results in strong competition and also increases the potential for cooperation, thus creating a difference between individual and group performance that can be utilized [8], [11]. As the planting density increases, the effective number of branches and pods per plant decrease, accompanied by the adjustment of yield components per unit area [8]–[9], [12]. It was reported that the transplanting of seedlings was commonly practiced in all kinds of agricultural systems and a relatively low yield was obtained only at the planting density of 10–15 plants m^-2^ in rapeseed [12]. However, in Europe, the optimal planting density was about 80–150 plants m^-2^ before winter and 60–80 plants m^-2^ at the beginning of spring, respectively [13]. Since mechanical production has been popularized along the Yangtze River, it's the need of time to reform the traditional rapeseed cultivation system by optimizing the planting density to have maximum seed yield of the crop.

Planting density influences the yield by regulating growth, yield components, and photosynthesis, which are the target traits closely related to the ideotype of crops [14]–[15]. The morphological ideotype traits of “super” rice were reflected in its moderate tillering capacity, heavy and drooping panicles, and a leaf area index (LAI) of ∼6.0 in the top three leaves [15]. The contributing traits and mechanisms suggest that an increase in total biomass accumulation, better partitioning efficiency, and sustained photosynthesis are the major physiological determinants of yield increases [16]–[18]. In rapeseed, the leaf is the photosynthetic source before anthesis, whereas it is the lower part of the plant after anthesis, which receives less radiation due to the development of the silique canopy, and the green silique wall photosynthesis during seed-filling contributes ∼2/3 of the total dry matter of the seeds [19]. Other studies have also highlighted the important role of silique wall photosynthesis in the regulation of seed oil content [20].

Few studies have described the ideal population structure in winter rapeseed. To provide useful information for high seed yield cultivation and breeding, and for the mechanical production as well, understanding the ideotype traits is necessary. The objectives of this study were to optimize the yield and yield components of two elite winter varieties that were commonly grown in the Yangtze River basin under several planting densities and to identify the physiological mechanisms that contribute to the high yield.

## Materials and Methods

### Experimental design

Two field trials were conducted in 2010–2013 at Yangluo Experimental Station of the Oil Crops Research Institute, Chinese Academy of Agricultural Science in Wuhan, Hubei Province, China (30°6′N, 114°1′E), which is located approximately in the center of the Yangtze River basin. The soil in the experimental field was representative of the area. The soil type was yellow-brown and soil samples were collected between 0 and 30 cm. Soil samples were air dried, ground, and analyzed for pH, dissolved organic carbon (DOC), total nitrogen, alkaline digested N, available phosphorus, available potassium, and available boron (S1 Table).

The first experiments which were conducted in 2010–2011 and 2011–2012 were aimed to study the effects of planting densities on seed yield and yield components. The sowing dates were on 25 September in 2010 and 2011, respectively. The conventional winter rapeseed variety Zhongshuang 11 (ZS11) and the hybrid variety Huayouza 9 (HYZ9) were used and planted with a split-plot design with three replicates. The main plots were established with five planting densities (27.0×10^4^, 37.5×10^4^, 48.0×10^4^, 58.5×10^4^, and 69.0×10^4^ plants ha^–1^) with the codes of D1–D5, and the subplots were varieties. In 2 m×10 m sized subplots and in rows about 30–35 cm apart (three rows per meter), the plants were finalized by hand when the seedlings had fully developed 4–5 true leaves and the spaces were ranged from 4 m to 11 m to achieve different planting densities. Each plot was fertilized at the average level in the Yangtze River basin with urea (195 kg ha^–1^ N), superphosphate (75 kg ha^–1^ P_2_O_5_), potassium chloride (105 kg ha^–1^ K_2_O), and borax (9 kg ha^–1^ boron). The nitrogen was applied in a split way, 60% at sowing and 40% at the seedling stage, whereas the phosphorus, potassium, and borax were all applied at sowing.

**Figure 1:**
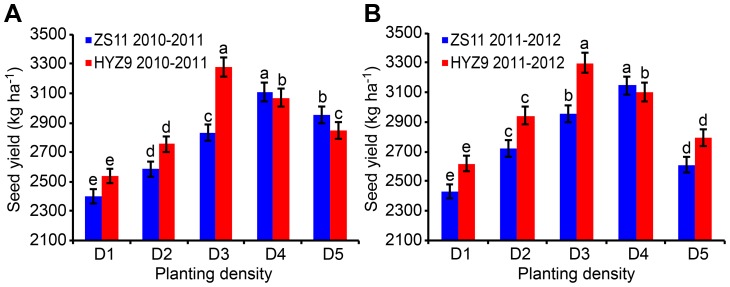
Seed yield of ZS11 and HYZ9 in 2010–2011 and 2011–2012 growing seasons. (A) The seed yield of ZS11 and HYZ9 in 2010–2011. (B) The seed yield of ZS11 and HYZ9 in 2011–2012. The planting densities were designed as D1, 27.0×10^4^ plants ha^-1^; D2, 37.5×10^4^ plants ha^-1^; D3, 48.0×10^4^ plants ha^-1^; D4, 58.5×10^4^ plants ha^-1^; D5, 69.0×10^4^ plants ha^-1^. Different lower case letters indicate significant pairwise differences between means (p<0.05; Duncan's test).

On the bases of the first experiment, the second experiment was conducted in 2011–2012 and 2012–2013 to study the physiological traits of different populations. As the relatively low seed yield was observed at a planting density of 27.0×10^4^ plants ha^–1^, it was referred as the traditionally normal yield population. However, the highest seed yield was obtained at increasing planting densities in the first experiment, 58.5×10^4^ plants ha^–1^ and 48.0×10^4^ plants ha^–1^ were referred as high yield population in ZS11 and HYZ9, respectively (Fig. 1). In 2012–2013, the seeds were sown on 28 September 2012 by using densities of high-yield and normal-yield populations, as described above. A randomized complete block design with three replicates was established, and 12 plots were designed for the two varieties. The plot area was 2 m long ×10 cm wide and consisted of 30 rows. A 1m wide border was left around each plot. The rates of application of N, P_2_O_5_, and K_2_O were the same as those in the first experiment.

### Data collection and analysis

#### Yield, yield components, and seed quality

At maturity, plants from an area of 1 m^2^ (1 m×1 m) of each plot were sampled to determine the yield components (i.e., the silique numbers, number of seeds per silique, and 1000-seed weights on the main inflorescences and branches), and the numbers of branches per plant and per unit area were counted as well. The plants in the plots were harvested, and the seed yields per unit area were calculated. The seed yield was adjusted to account for 9% moisture content. We measured the seed oil content from the main inflorescences, branches, and each plot following the Chinese National Standard Method for the determination of the oil content in oilseeds (GB/T 14488.1-93).

#### Leaf area index (LAI), silique wall area index (SAI), and photosynthesis

At 90, 120, 150, 180, and 210 days after sowing as well as at 7-day intervals after peak anthesis, the measurement of leaf area index (LAI) and a gas exchange analysis were conducted. The green leaf area was measured by passing the leaves through a LI-3100 leaf area meter (LiCor, Lincoln, NE, USA). At the seed-filling stage, the silique wall area index (SAI) and silique wall photosynthesis were measured at 7-day intervals after peak anthesis. Fifty siliques on the main inflorescences and all of the branches were randomly sampled to measure the silique length and width, and the silique wall area was calculated according to [21]. The LAI and SAI were then determined on a ground area basis.

The photosynthetic parameters were determined on the fifth leaves at 90 days after sowing and on the first short petiole leave of the plants from 120 to 210 days after sowing in the high-yield and normal-yield population. The gas exchange analysis was conducted using a Portable Photosynthesis System (LI-6400; LiCor) on the leaves and pods from 09:00 to 11:00. The net photosynthetic rates (Pn), stomatal conductance (Gs), intercellular CO_2_ concentration (Ci), and transpiration rate (Tr) were determined. Water use efficiency (WUE) was calculated as the ratio of Pn to Tr. The data were collected automatically every 2–3 min with 10 replications for each plot.

#### Dry matter accumulation

All the plants per unit area (1 m^2^) in each normal-yield and high-yield population were selected, and the aerial parts were collected at 2 weeks pre-anthesis and 2 weeks post-anthesis. All of the aerial parts were dried at 105°C for 30 min and then at 70°C to constant weight. At maturity, the stems, silique wall and seeds at the same squares were separated and dried at 70°C to constant weight.

#### Statistical analysis

We performed multi-way ANOVAs with critical values of *p* = 0.05 using Statistix 8. Significant pairwise differences between means were identified by Duncan's multiple range test (*p*<0.05) using SPSS software (version 16.0; SPSS Inc., Chicago, IL, USA). The correlation analysis between the seed yield with the yield components (e.g., silique number and number of primary branches per unit area), with the growth parameters (e.g., LAI, SAI and dry matter biomass) as well as with the physiological traits (e.g., leaf photosynthesis and silique wall photosynthesis) were carried out.

## Results

### Yield components of main inflorescences and branches

Planting density did not affect the number of siliques on the main inflorescences, seeds per silique, or 1000-seed weight in ZS11 and HYZ9 in either year. In comparison of the growing seasons, the numbers of siliques and seeds per silique were significantly higher in 2011–2012 than in 2010–2011 (S2 Table). The experimental treatments had more pronounced effects on the yield components of branches than on the main inflorescences. As shown in Table 1, at the individual level, the number of primary branches decreased significantly with an increase in planting densities in both seasons. The lowest number of primary branches was observed at the highest planting density (D5). Increasing the planting density also dramatically decreased the number of siliques and the seeds per silique. However, the 1000-seed weight was not affected. It was notably that the 1000-seed weight of the main inflorescences was 1.0–1.5 g higher than that of the branches. The ANOVAs results showed that the yield components were obviously affected not only by the year, variety and planting density but also by their interactions. The number of primary branches, silique number and seed per silique varied significantly for interactions between any two of year, variety and planting density. However, no significant differences were observed for 1000-seed weight.

**Table 1:** Yield components of branches in ZS11 and HYZ9 in 2010–2011 and 2011–2012 growing seasons.

Variety	Planting density (×10^4^ plants ha^-1^)	Number of primary branches (n plant^-1^)	Silique numbers (n plant^-1^)	Seeds per silique	1000-seed weight (g)
2010–2011					
ZS11	27.0	7.3a	152.1a	18.4a	3.77a
	37.5	6.0b	128.8b	16.2b	3.55a
	48.0	5.0c	102.8c	15.8b	3.55a
	58.5	4.4d	88.5d	15.2c	3.28a
	69.0	3.2e	50.2e	14.0d	3.37a
HYZ9	27.0	7.6a	204.4a	18.9a	3.33a
	37.5	6.8b	168.3b	18.1b	3.22a
	48.0	6.2b	138.4c	17.2c	3.20a
	58.5	5.0c	92.0d	16.0d	3.08a
	69.0	4.2d	62.8e	14.3e	2.92a
2011–2012					
ZS11	27.0	7.9a	177.4a	19.3a	3.89a
	37.5	6.4b	151.6b	19.5a	3.66a
	48.0	5.3c	117.8c	17.6b	3.65a
	58.5	4.5d	88.9d	15.9c	3.37a
	69.0	3.5d	59.7e	15.6c	3.32a
HYZ9	27.0	8.1a	202.0a	19.9a	3.32a
	37.5	6.7b	182.5b	19.2a	3.23a
	48.0	6.4b	150.3c	18.2b	3.20a
	58.5	5.2c	99.0d	16.3c	3.11a
	69.0	4.4d	65.4e	15.8c	3.00a
Year (Y)		*	**	**	^†^NS
Variety (V)		*	**	**	^†^NS
Year (Y) × variety (V)		**	*	**	^†^NS
Year (Y) × density (D)		**	**	**	^†^NS
V×D		**	**	**	^†^NS
Y×V×D		**	**	**	^†^NS

Different letters within the same column indicate significant differences between the means determined by Duncan's multiple range test (*p*<0.05). For the interaction terms: *, ** show the significance at 0.05 and 0.01 levels, ^†^NS not significant.

### Silique numbers, primary branches, and yields per unit area

**Figure 2:**
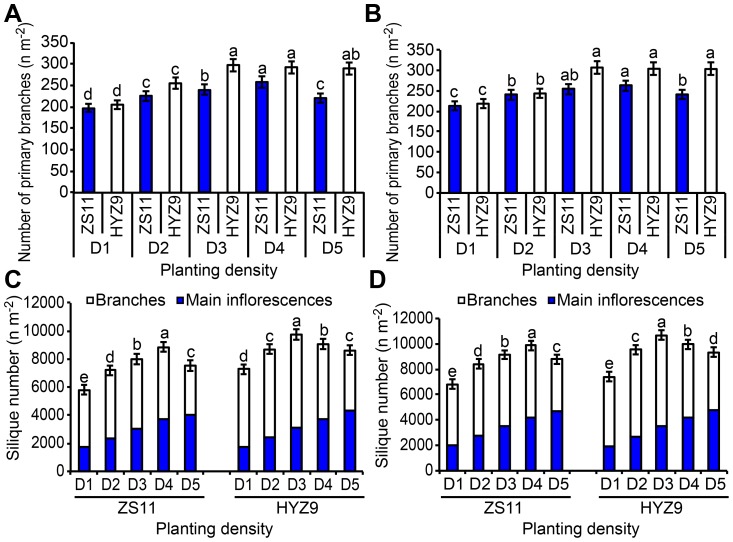
Numbers of primary branches and siliques per unit area of ZS11 and HYZ9 at five planting densities in 2010–2011 and 2011–2012 growing seasons. (A) and (B) Number of primary branches per unit area of ZS11 and HYZ9 in 2010–2011 and 2011–2012, respectively. (C) and (D) Number of siliques per unit area of ZS11 and HYZ9 in 2010–2011 and 2011–2012, respectively. Different lower case letters indicate significant pairwise differences between means (p<0.05; Duncan's test).

The number of primary branches per unit area initially increased as the planting density increased and then decreased rapidly in ZS11. The maximum number of branches was observed at the planting density D4. The results also indicated that the highest number of primary branches occurred at the planting density D3 and decreased steadily as the planting density increased in HYZ9 (Fig. 2A and B). The number of siliques on the main inflorescences increased with increasing planting density in both years (Fig. 2C and D), whereas those on branches initially increased with increasing planting density and then decreased significantly at the highest planting density in both varieties. The maximum number of siliques was observed at the densities of D4 and D3 in ZS11 and HYZ9, respectively. Consequently, the seed yields per unit area were also initially positively affected and then negatively affected by increasing the planting density in both years. In 2010–2011, the seed yield at the planting density D4 was 29.5% higher than at the planting density D1 in ZS11, and the seed yield at D3 was 29.2% higher than that at D1 in HYZ9 (Fig. 1A). In 2011–2012, the planting density D4 resulted in a 26.7% higher seed yield than D1 in ZS11, and D3 gave a 25.9% higher seed yield than D1 in HYZ9 (Fig. 1B). The number of primary branches and the total number of silique per unit area were extremely significantly correlated with the seed yield (r = 0.7754**and r = 0.8524**, respectively).

Neither the seed oil content of the main inflorescences nor branches displayed significant differences among the different planting densities, whereas the seed oil content from the main inflorescences was 1.0–2.5% higher than from the branches. Notably, the oil content per plot increased significantly with increased planting density (S3 Table).

### LAI, SAI, and photosynthesis

**Figure 3:**
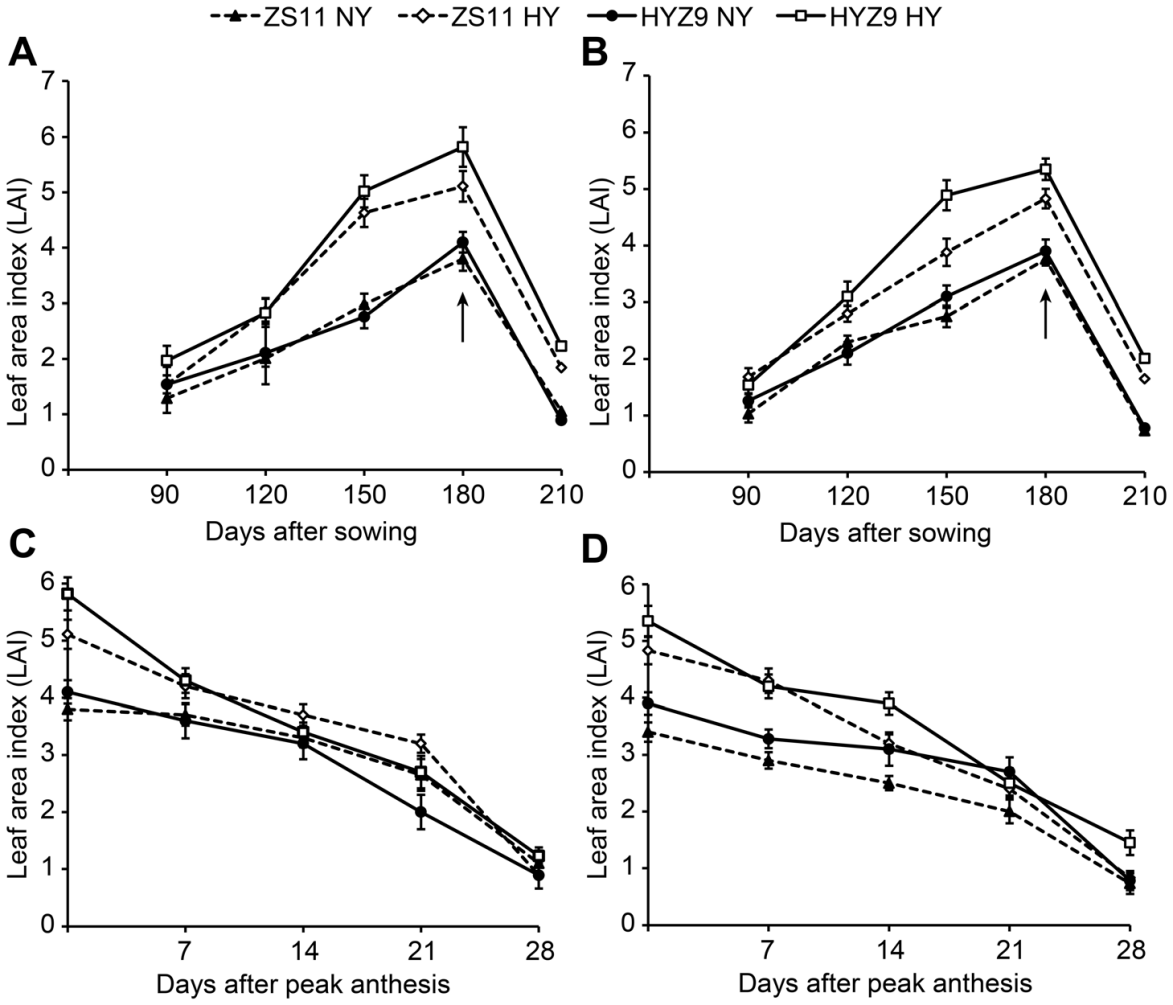
Leaf area index (LAI) at different growing stages and post-anthesis in the normal-yield (NY) and high-yield (HY) populations of ZS11 and HYZ9 in 2011–2012 and 2012–2013 growing seasons. (A) and (B) LAI at different growing stages in the NY and HY populations of ZS11 and HYZ9 in 2011–2012 and 2012–2013, respectively. (C) and (D) LAI at post-anthesis in the NY and HY populations of ZS11 and HYZ9 in 2011–2012 and 2012–2013, respectively. The arrows indicate the flowering stage.

The LAI increased from 90 to 180 days after sowing and then decreased. The values were higher in the high-yield population than in the normal-yield population, and in 2011–2012, the maximum values of 5.11 and 5.82 were recorded 180 days after sowing in ZS11 and HYZ9, respectively (Fig. 3A). In 2012–2013, the maximum values of 4.83 and 5.35 were obtained in ZS11 and HYZ9, respectively (Fig. 3B). The LAI decreased rapidly in the high-yield population after anthesis, and no apparent difference was observed after 14 days of the peak anthesis between the two populations in either year (Fig. 3C and D).

**Figure 4:**
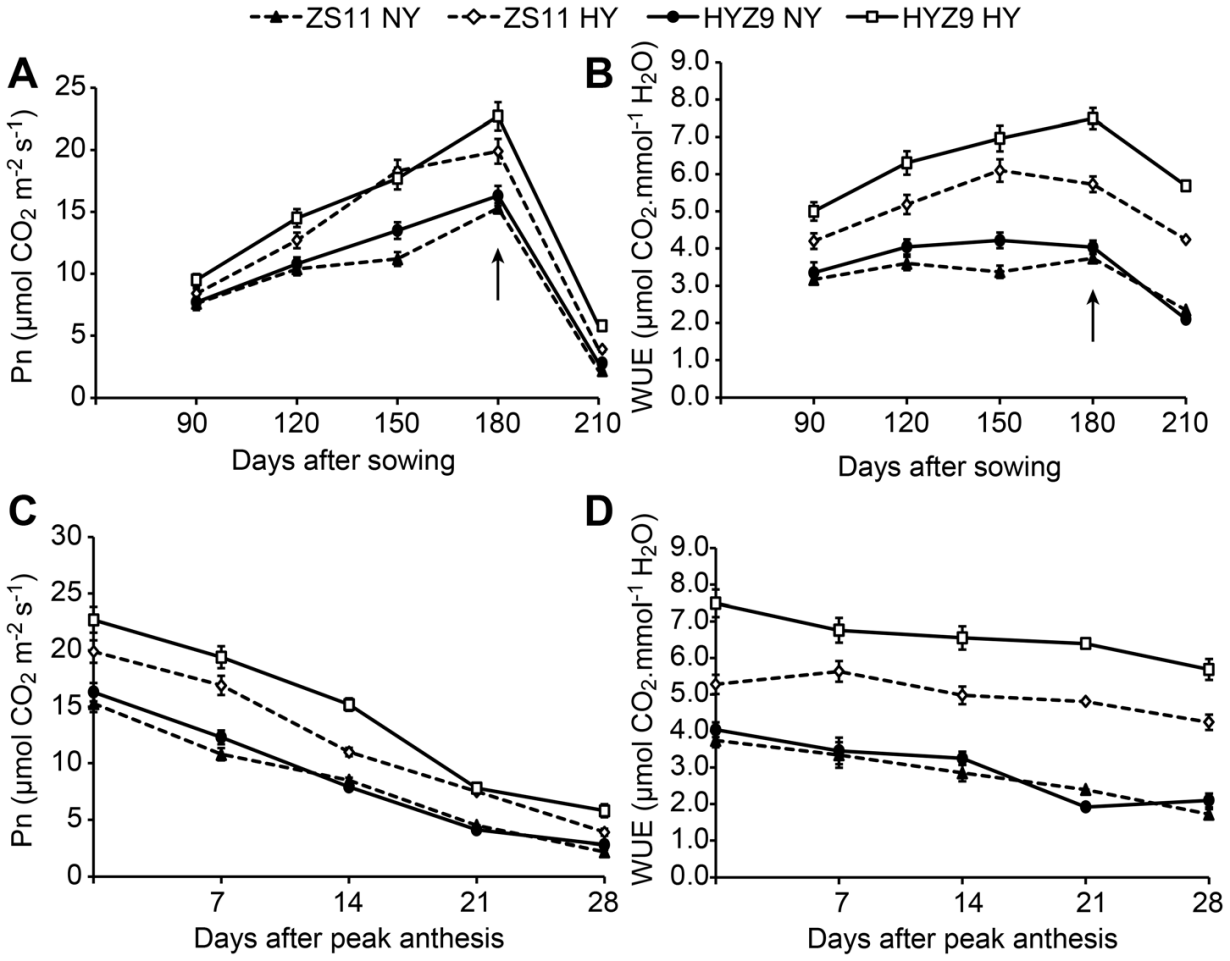
The net photosynthetic rates (Pn), and water use efficiency (WUE) of leaves at different growing stages and post-anthesis in the normal-yield (NY) and high-yield (HY) populations of ZS11 and HYZ9 in 2011–2012 growing season. (A) and (B) Pn and WUE of leaves at different growing stages in the NY and HY populations of ZS11 and HYZ9, respectively. (C) and (D) Pn and WUE of leaves at post-anthesis in the NY and HY populations of ZS11 and HYZ9, respectively. The arrows indicate the flowering stage.

**Figure 5:**
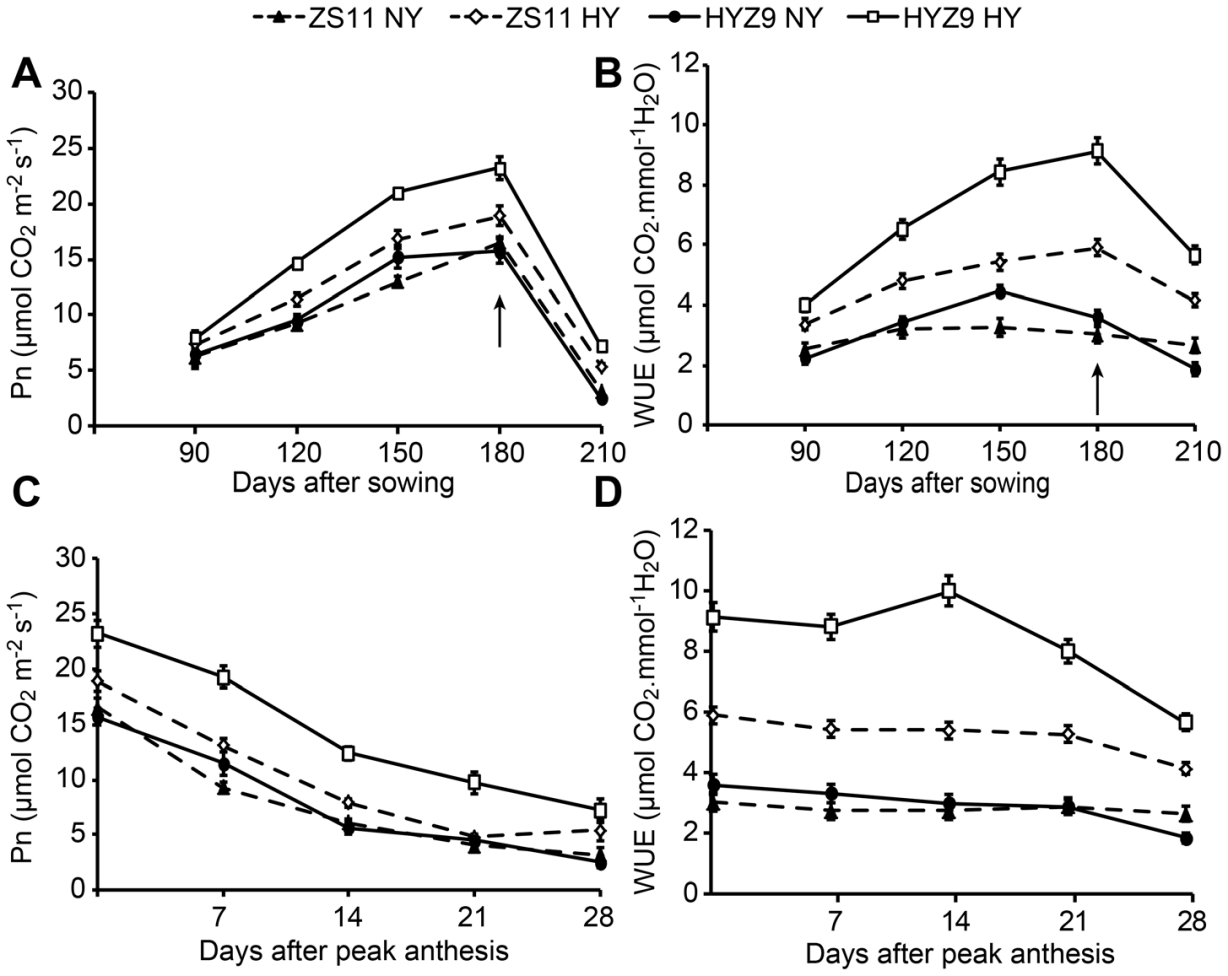
The net photosynthetic rates (Pn), and water use efficiency (WUE) of leaves at different growing stages and post-anthesis in the normal-yield (NY) and high-yield (HY) populations of ZS11 and HYZ9 in 2012–2013 growing season. (A) and (B) Pn and WUE of leaves at different growing stages in the NY and HY populations of ZS11 and HYZ9, respectively. (C) and (D) Pn and WUE of leaves at post-anthesis in the NY and HY populations of ZS11 and HYZ9, respectively. The arrows indicate the flowering stage.

In both seasons, the net photosynthetic rate (Pn), stomatal conductance (Gs), and intercellular CO_2_ concentration (Ci) of leaves increased rapidly from 150 to 180 days after sowing in the high-yield population. However, the transpiration rate (Tr) was significantly lower (data not shown), which resulted in a higher water use efficiency (WUE) (Fig. 4A and B; Fig. 5A and B). Pn decreased rapidly 14 days after peak anthesis in the high-yield population, and the WUE was higher but decreased rapidly as well (Fig. 4C and D; Fig. 5C and D).

**Figure 6:**
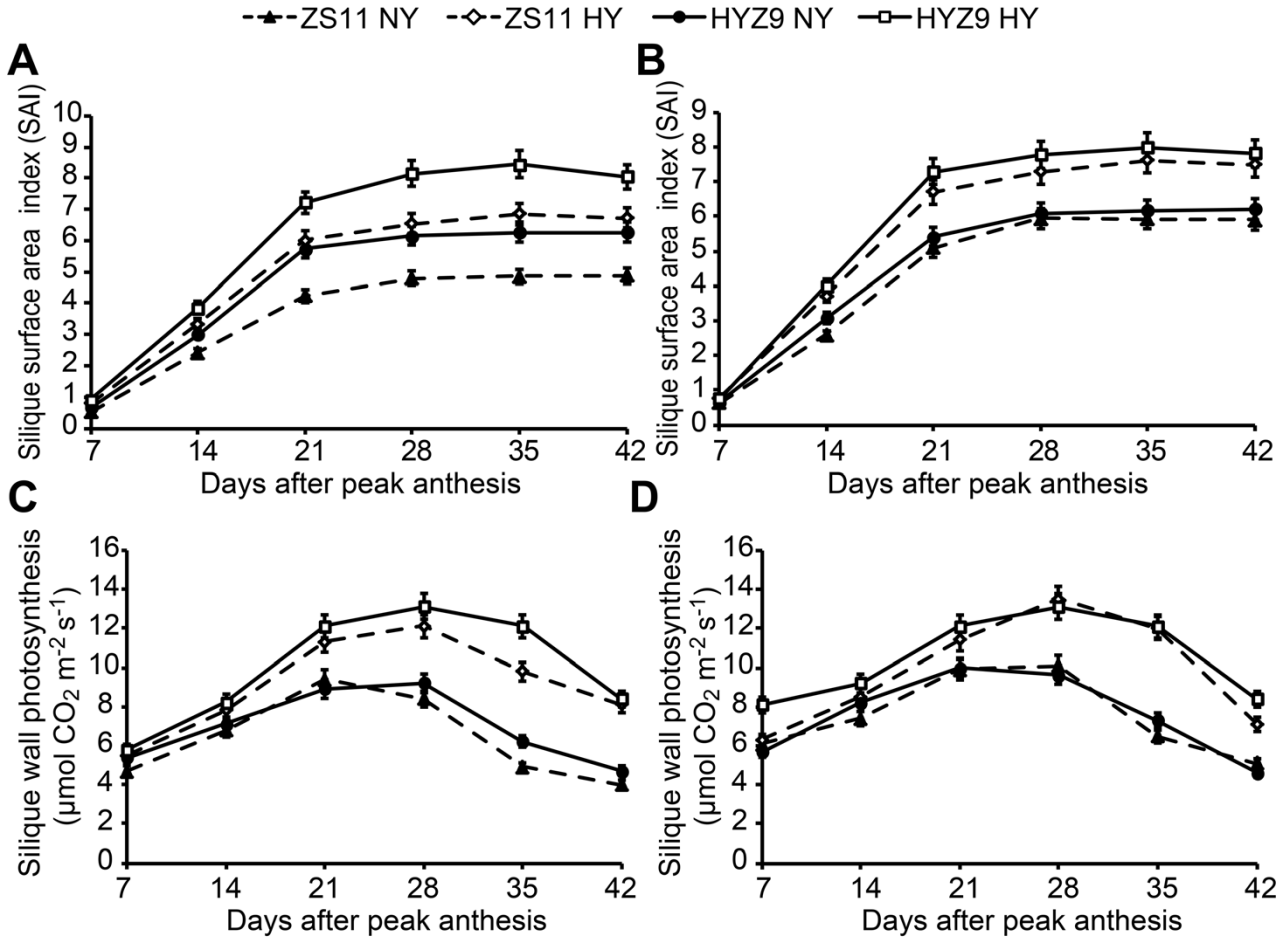
The silique wall area index (SAI) and silique wall photosynthesis in the normal-yield (NY) and high-yield (HY) populations of ZS11 and HYZ9 in 2011–2012 and 2012–2013 growing seasons. (A) and (B) SAI in the NY and HY populations of ZS11 and HYZ9 in 2011–2012 and 2012–2013, respectively. (C) and (D) The silique wall photosynthesis in the NY and HY populations of ZS11 and HYZ9 in 2011–2012 and 2012–2013, respectively.

Within all the populations, the SAI increased rapidly from 7 to 21 days and reached a maximum ∼28 days after peak anthesis. Apparent difference was observed between the high-yield and normal-yield populations from 21 to 42 days after peak anthesis in either variety (Fig. 6A and B). The change trend of silique wall photosynthesis was similar to that of the SAI from 7 to 28 days, and the high-yield populations had longer duration of high photosynthetic rates from 21 to 35 days in both varieties (Fig. 6C and D).

Not only LAI at peak anthesis, but also the leaf photosynthesis, SAI and silique wall photosynthesis showed significant correlations with the seed yield (r = 0.9583**, r = 0.9338**, r = 0.9100** and r = 0.9541**, respectively).

### Dry matter accumulation

**Figure 7:**
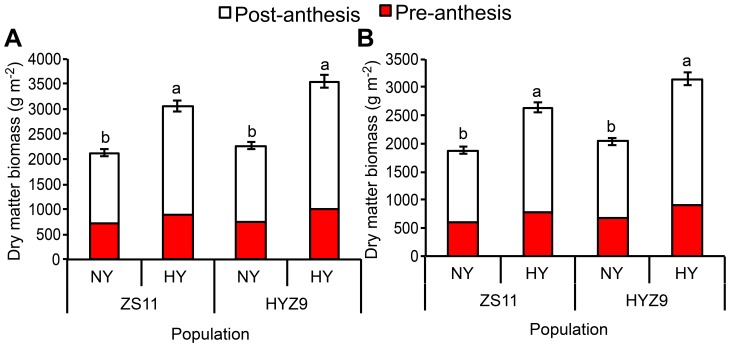
Dry matter biomass at pre-anthesis and post-anthesis in the normal-yield (NY) and high-yield (HY) populations of ZS11 and HYZ9 in 2011–2012 and 2012–2013 growing seasons. (A) Dry matter biomass at pre-anthesis and post-anthesis in the NY and HY populations of ZS11 and HYZ9 in 2011–2012. (B) Dry matter biomass at pre-anthesis and post-anthesis in the NY and HY populations of ZS11 and HYZ9 in 2012–2013. Different lower case letters indicate significant pairwise differences between means (p<0.05; Duncan's test).

For the dry matter biomass at 2 weeks pre-anthesis and 2 weeks post-anthesis (Fig. 7), the values of dry matter per unit area at pre-anthesis were 706 g m^–2^ and 903.5 g m^–2^, and 1416.1 g m^–2^ and 2154.3 g m^–2^ at post-anthesis in the normal- and high-yield ZS11 populations in 2011–2012, respectively, with the ratios of pre-anthesis to post-anthesis dry matter 0.50 and 0.42. In the normal- and high-yield HYZ9 populations, the values of dry matter per unit area were 742.9 g m^–2^ and 1002.5 g m^–2^ at pre-anthesis, and 1522.1 g m^–2^ and 2539.5 g m^–2^ at post-anthesis, respectively, having the pre-anthesis to post-anthesis dry matter ratios of 0.49 and 0.39.

For the normal- and high-yield ZS11 populations in 2012–2013, the values of dry matter per unit area were 601 g m^–2^ and 774 g m^–2^ at pre-anthesis and 1277.5 g m^–2^ and 1865.6 g m^–2^ at post-anthesis, respectively, showing the pre-anthesis to post-anthesis dry matter ratios of 0.47 and 0.41. In the two HYZ9 populations, the values of dry matter per unit area were 675 g m^–2^ and 906 g m^–2^ at pre-anthesis and1365.4 g m^–2^ and 2243.8 g m^–2^ at post-anthesis, respectively, with the pre-anthesis to post-anthesis ratios of 0.49 and 0.40. The dry matter weights at pre-anthesis and post-anthesis were extremely significantly correlated with seed yield (r = 0.8739**and r = 0.9336**, respectively).

## Discussion

Yield improvement can be achieved by adjusting elements of the growing system, such as the planting density [6], [9], [13], [22]. The results of this study indicate that variations in the planting density can significantly affect the seed yield and yield components of winter rapeseed. Moreover, this study is also showing that planting density has great effects on the yield components of primary branches than the main inflorescences. The number of siliques per plant and the number of siliques per unit area are known to be the most variable and dominant yield components [8], [23]–[25]. In this study, the highest seed yield was associated with the highest number of primary branches and the total number of siliques per unit area at the planting densities of 58.5×10^4^ plants ha^–1^ and 48.0×10^4^ plants ha^–1^ in ZS11 and HYZ9, respectively, which are much higher than the planting densities currently used in large-scale cultivation in China.

The seed oil content of the main inflorescences and branches increased only slightly and insignificantly at the higher planting density, whereas the content per plot increased significantly. These results contrasted the others [12], possibly owing to the facts that the seed oil content of main inflorescences was 1.0–2.5% higher than that of branches and the proportion of yield accounted for by the main inflorescence rising with increased planting density from 35 to 60% (data not shown). Furthermore, with the increase in planting densities, the production of pod-bearing branches decreased and the development of pods and seeds were synchronized, which might result in more uniform maturation and higher seed quality [23]. Taken together, seed yield and oil content could be simultaneously improved at relatively high planting densities in the modern cultivation system.

Physiological traits could directly contribute to higher yields [26]. The planting density influences the crop canopy structure and radiation interception [27]. The population with a high LAI is likely to intercept light because radiation is better distributed throughout the canopy, thus resulting in higher use efficiency [10],[18]. In our study, the highest LAI was ∼5.0 at the high planting density, which suggests that the plants grow rapidly at high planting densities and provide a larger photosynthetic area. A significantly higher Pn and WUE were observed at the higher planting density from 150 to 180 days after sowing in both varieties. The rapid decrease in LAI and Pn in the high-yield population after anthesis was likely due to the high amount of translocation of carbohydrates from vegetative to reproductive organs, which indicates early leaf senescence. The postponement of silique wall photosynthesis senescence was a consequence of the better radiation distribution throughout the canopy with moderate number of siliques that comprised the optimal number of main inflorescences and branches. It is suggested that the high silique wall photosynthetic rate and its longer duration are likely to promote dry matter accumulation and be responsible for the increase in yield and oil content [16], [18], [20]. Taken together, the high seed yield highlights the successful alteration of high leaf photosynthesis before anthesis and longer duration of high silique wall photosynthesis after peak anthesis.

To better understand the plant growth contributing to the high seed yield, the dry matter biomass was investigated, for many studies have indicated that the high yield was associated with dry matter production before [16], [28] and after heading [29]–[31]. In our study, the high-yield populations had high dry matter biomass at both pre-anthesis and post-anthesis, with the latter likely largely contributes to yield increase. Therefore, it is supposed that high planting densities promote the development of vegetative organs before anthesis and effective nutrition uptake dynamics to the reproductive organs after anthesis, which strongly impact the increase of seed yield.

The design of plant ideotype is an important step toward improving yields [32]. The ideotypes of some cereal crops, such as rice and maize were simulated [8], [33]. Meanwhile, an ideotype may require a degree of crop management to fully express its yield potential [33]–[34]. It is proposed that the maize (*Zea mays* L.) ideotype will maximally utilize an optimal production environment including high planting densities and be characterized by maximum photosynthetic efficiency and efficient conversion of photosynthate to grain [33]. In our study, the ideal morphological and physiological traits (LAI, SAI, photosynthesis, WUE) of rapeseed population were observed at the higher planting density, which would greatly benefit the mechanical production and yield potential.

## Conclusions

To explore the ideotype population of winter rapeseed in the central part of the Yangtze River basin, the yield components, morphological and physiological traits of two elite cultivars were investigated under field conditions. The results showed that the planting density had no significant effect on the yield components of the main inflorescences but it clearly affected the branch number, silique number, and seeds per silique on the branches per plant. The ideal morphological traits of the two varieties were moderate number of siliques and primary branches per plant as well as high number of siliques and primary branches per unit area. Furthermore, higher LAI (∼5.0), Pn, and WUE were observed in the high-yield population, whereas they decreased more rapidly after anthesis compared with the normal-yield population. It was suggested that the higher SAI (∼7.0) and longer duration of high silique wall photosynthesis likely resulted in a significantly higher biomass at the seed-filling stage and a subsequently higher seed yield. Wholly, the improvement of the seed yield and oil content of winter rapeseed is possible by managing higher planting density and ideotype population.

## Supporting Information

S1 Table Soil properties at the beginning of the 2010–2011, 2011–2012, and 2012–2013 growing seasons.
(DOC)

S2 Table Yield components of the main inflorescences in ZS11 and HYZ9 in 2010–2011 and 2011–2012 growing seasons.
(DOC)

S3 Table Seed oil content of ZS11 and HYZ9 in 2010–2011 and 2011–2012 growing seasons.
(DOC)
