# Supplementary material for: Ideotype Population Exploration: Growth, Photosynthesis, and Yield Components at Different Planting Densities in Winter Oilseed Rape (Brassica napus L.)
Source: PLoS One. 2014 Dec 17;9(12):e114232. doi: 10.1371/journal.pone.0114232 (PMC4269386; doi:10.1371/journal.pone.0114232)
Supplement: S1 Table — Soil properties at the beginning of the 2010–2011, 2011–2012, and 2012–2013 growing seasons. (DOC) [file pone.0114232.s001.doc]

**Table S1** Soil properties at the beginning of the 2010–2011, 2011–2012, and 2012–2013 growing seasons.

| Parameter | Unit | 2010–2011 | 2011–2012 | 2012–2013 |
| --- | --- | --- | --- | --- |
| pH |  | 6.65 | 6.82 | 6.70 |
| Dissolved organic carbon | mg kg^-1^ | 85.3 | 95.1 | 104.1 |
| Total N | g kg^-1^ | 1.51 | 1.69 | 1.79 |
| Alkaline digested N | mg kg^-1^ | 74.2 | 78.2 | 79.3 |
| Available phosphorus | mg kg^-1^ | 46.2 | 45.7 | 50.5 |
| Available potassium | mg kg^-1^ | 63.1 | 60.3 | 68.4 |
| Available B | mg kg^-1^ | 0.45 | 0.51 | 0.66 |
