# Supplementary material for: Ideotype Population Exploration: Growth, Photosynthesis, and Yield Components at Different Planting Densities in Winter Oilseed Rape (Brassica napus L.)
Source: PLoS One. 2014 Dec 17;9(12):e114232. doi: 10.1371/journal.pone.0114232 (PMC4269386; doi:10.1371/journal.pone.0114232)
Supplement: S2 Table — Yield components of the main inflorescences in ZS11 and HYZ9 in 2010–2011 and 2011–2012 growing seasons. (DOC) [file pone.0114232.s002.doc]

**Table S2** Yield components of the main inflorescences in ZS11 and HYZ9 in 2010–2011 and 2011–2012 growing seasons.

| Variety | Planting density (×10^4^ plants ha^-1^) | Silique numbers (n plant^-1^) | Seeds per silique | 1000-seed weight (g) |
| --- | --- | --- | --- | --- |
| 2010–2011 |  |  |  |  |
| ZS11 | 27.0 | 63.3a | 18.2a | 4.81a |
|  | 37.5 | 63.2a | 18.3a | 4.87a |
|  | 48.0 | 63.8a | 18.5a | 4.89a |
|  | 58.5 | 63.0a | 17.8a | 4.88a |
|  | 69.0 | 58.8a | 17.2a | 4.53a |
| HYZ9 | 27.0 | 65.4a | 20.8a | 4.53a |
|  | 37.5 | 64.2a | 19.7a | 4.56a |
|  | 48.0 | 64.7a | 19.2a | 4.42a |
|  | 58.5 | 63.2a | 19.6a | 4.38a |
|  | 69.0 | 62.3a | 19.1a | 4.43a |
| 2011–2012 |  |  |  |  |
| ZS11 | 27.0 | 74.9a | 20.6a | 5.05a |
|  | 37.5 | 73.3a | 19.5a | 5.11a |
|  | 48.0 | 72.8a | 19.3a | 4.88a |
|  | 58.5 | 71.3a | 19.6a | 4.95a |
|  | 69.0 | 67.8a | 19.2a | 4.72a |
| HYZ9 | 27.0 | 72.5a | 21.1a | 4.69a |
|  | 37.5 | 72.2a | 20.4a | 4.64a |
|  | 48.0 | 72.7a | 20.1a | 4.65a |
|  | 58.5 | 71.4a | 19.9a | 4.53a |
|  | 69.0 | 69.8a | 19.0a | 4.50a |
| Year(Y) | | ** | ** | ^†^NS |
| Variety(V) | | ^†^NS | ^†^NS | ^†^NS |
| Year(Y)×Variety(V) | | ^†^NS | ^†^NS | ^†^NS |
| Year(Y)×density(D) | | ^†^NS | ^†^NS | ^†^NS |
| V×D | | ^†^NS | ^†^NS | ^†^NS |
| Y×V×D | | ^†^NS | ^†^NS | ^†^NS |

Means for one-way terms within the same column were analyzed according to linear models in Statistix 8, and those with significant differences determined by Duncan's multiple range test (*p*<0.05) are followed by different letters. For the interaction terms: ^†^NS, not significant; ^**^Significant at the 0.01 level.
