# Supplementary material for: Ideotype Population Exploration: Growth, Photosynthesis, and Yield Components at Different Planting Densities in Winter Oilseed Rape (Brassica napus L.)
Source: PLoS One. 2014 Dec 17;9(12):e114232. doi: 10.1371/journal.pone.0114232 (PMC4269386; doi:10.1371/journal.pone.0114232)
Supplement: S3 Table — Seed oil content of ZS11 and HYZ9 in 2010–2011 and 2011–2012 growing seasons. (DOC) [file pone.0114232.s003.doc]

**Table S3** Seed oil content of ZS11 and HYZ9 in 2010–2011 and 2011–2012 growing seasons.

| Variety | Planting density (×10^4^ plants ha^-1^) | Oil content (%) | | |
| --- | --- | --- | --- | --- |
|  |  | Main inflorescences | Branches | plot |
| 2010–2011 |  |  |  |  |
| ZS11 | 27.0 | 45.76a | 44.42a | 44.15d |
|  | 37.5 | 45.80a | 44.77a | 45.13c |
|  | 48.0 | 46.77a | 45.13a | 46.32b |
|  | 58.5 | 47.09a | 44.39a | 46.77b |
|  | 69.0 | 47.25a | 45.28a | 47.74a |
| HYZ9 | 27.0 | 43.87a | 42.43a | 42.23c |
|  | 37.5 | 44.67a | 42.48a | 42.65c |
|  | 48.0 | 44.47a | 42.55a | 43.89b |
|  | 58.5 | 44.08a | 42.70a | 44.15b |
|  | 69.0 | 45.25a | 42.77a | 45.32a |
| 2011–2012 |  |  |  |  |
| ZS11 | 27.0 | 46.26a | 44.04a | 44.46d |
|  | 37.5 | 46.68a | 44.88a | 45.64c |
|  | 48.0 | 47.55a | 45.19a | 46.59b |
|  | 58.5 | 47.06a | 44.89a | 46.80b |
|  | 69.0 | 47.61a | 45.37a | 48.35a |
| HYZ9 | 27.0 | 43.89a | 42.19a | 42.33c |
|  | 37.5 | 44.09a | 42.23a | 42.37c |
|  | 48.0 | 44.54a | 43.09a | 43.41b |
|  | 58.5 | 44.85a | 43.03a | 44.38a |
|  | 69.0 | 45.37a | 43.24a | 44.74a |
| Year(Y) | | ** | ** | ** |
| Variety(V) | | ** | ** | ** |
| Year(Y)×Variety(V) | | ** | ** | ** |
| Year(Y)×density(D) | | ** | ** | ** |
| V×D | | ** | ** | ** |
| Y×V×D | | ** | ** | ** |

Means for one-way terms within the same column were analyzed according to linear models of Statistix 8, and those with significant differences determined by Duncan's multiple range test (*p*<0.05) are followed by different letters. For the interaction terms: ^**^Significant at the 0.01 level.
